# Draft Genome Sequence of *Photorhabdus luminescens* Strain DSPV002N Isolated from Santa Fe, Argentina

**DOI:** 10.1128/genomeA.00744-16

**Published:** 2016-07-28

**Authors:** Leopoldo Palma, Eleodoro E. Del Valle, Laureano Frizzo, Colin Berry, Primitivo Caballero

**Affiliations:** a Centro de Investigaciones y Transferencia de Villa María (CITVM-CONICET), Universidad Nacional de Villa María, Córdoba, Argentina; b Consejo Nacional de Investigaciones Científicas y Técnicas, Buenos Aires, Argentina; c Facultad de Ciencias Agrarias, Universidad Nacional del Litoral, Esperanza, Santa Fe, Argentina; d ICIVET (CONICET-UNL)-Departamento de Salud Pública, Facultad de Ciencias Veterinarias, Universidad Nacional del Litoral, Esperanza, Santa Fe, Argentina; e Cardiff School of Biosciences, Cardiff University, Cardiff, Wales, United Kingdom; f Instituto de Agrobiotecnología, CSIC, UPNA-Gobierno de Navarra, Campus Arrosadía, Mutilva Baja, Navarra, Spain; g Grupo de Protección de Cultivos, Departamento de Producción Agraria, Escuela Técnica Superior de Ingenieros Agrónomos, Universidad Pública de Navarra, Pamplona, Navarra, Spain

## Abstract

Here, we report the draft genome sequence of *Photorhabdus luminescens* strain DSPV002N, which consists of 177 contig sequences accounting for 5,518,143 bp, with a G+C content of 42.3% and 4,701 predicted protein-coding genes (CDSs). From these, 27 CDSs exhibited significant similarity with insecticidal toxin proteins from *Photorhabdus luminescens* subsp. *laumondii* TT01.

## GENOME ANNOUNCEMENT

The entomoparasitic nematode *Heterorhabditis bacteriophora* is used as a biological control agent against insect pests in agriculture. However, this nematode species is capable of killing insects by vectoring the symbiotic and broad-spectrum entomopathogenic bacterium *Photorhabdus luminescens* into the hemocoel of the insect host. This bacterium commonly resides in the *H. bacteriophora* gut during the symbiotic phase of the life cycle*.* During this stage, the infective juveniles (IJs) search for an insect host and penetrate its cuticle or natural openings (1). Once inside the hemocoel, the nematode regurgitates the bacterium, which rapidly proliferates and releases a number of insecticidal toxins that produce fast killing of the insect by a combination of both generalized toxemia and septicemia (1). In addition, this bacterium also synthesizes other peptides with potential biotechnological and pharmaceutical applications (enzymes, antibiotics, etc.) intended to overcome the activity and proliferation of other opportunistic microorganisms (2). In this work, we report the draft genome sequence of *P. luminescens* strain DSPV002N, which was isolated from hemolymph of dead *Galleria mellonella* larvae from Santa Fe, Argentina (3). Purified genomic DNA was obtained using the Wizard genomic DNA purification kit (Promega), according to the instructions for DNA isolation from Gram-negative bacteria. Genome sequencing was performed at the Wellcome Trust Centre for Human Genetics (London, United Kingdom) using high-throughput Illumina sequencing technology. Gene prediction and annotation were performed with the NCBI Prokaryotic Genome Annotation Pipeline (2016 release), although the genome was also analyzed with BLAST using an updated (custom) insecticidal toxin database containing known toxins from Gram-positive and Gram-negative entomopathogenic bacteria. The Illumina raw reads were trimmed and assembled into 177 contigs totaling 5,518,143 bp, with a G+C content of 42.3% and containing 4,701 predicted protein-coding genes (CDSs) plus 84 RNA genes. From them, 27 CDSs exhibited significant similarity with insecticidal toxins from *Photorhabdus luminescens* subsp. *laumondii* strain TT01 (2), namely, 15 insecticidal toxin complex proteins (TccA1, TccA3, TcaC, TccB1, TccB3, TccC, TcdA2, two similar TcdB1, TccC2, TccC3, TccC5, TccC6, TcdA5, and TcaZ), two PirB homolog toxins, and 10 additional CDSs with similarity against other putative insecticidal toxins.

### Nucleotide sequence accession numbers.

This whole-genome shotgun project has been deposited at DDBJ/EMBL/GenBank under the accession no. LWGQ00000000. The version described in this paper is version LWGQ00000000.1.
